# Supplementary material for: Mechanisms governing the pioneering and redistribution capabilities of the non-classical pioneer PU.1
Source: Nat Commun. 2020 Jan 21;11:402. doi: 10.1038/s41467-019-13960-2 (PMC6972792; doi:10.1038/s41467-019-13960-2)
Supplement: Supplementary file 7 — Source data [file 41467_2019_13960_MOESM7_ESM.zip › Source_Data/FigureS2/FigureS2B_MotifScanOutput/KmeansCluster6/homerResults/motif11.similar.html]

motif11

## Information for motif11

G
T
C
A
A
G
T
C
G
T
C
A
G
T
A
C
T
C
G
A
G
T
A
C
A
C
G
T
A
G
C
T
A
C
G
T
G
A
C
T
T
C
G
A
C
G
T
A
G
C
T
A
G
T
A
C
  
Reverse Opposite:  

C
A
T
G
C
A
G
T
G
A
C
T
A
C
G
T
C
T
G
A
T
C
G
A
C
T
G
A
T
G
C
A
A
C
T
G
A
C
G
T
C
A
T
G
A
C
G
T
C
T
A
G
A
C
G
T
  

|  |  |
| --- | --- |
| p-value: | 1e-66 |
| log p-value: | -1.531e+02 |
| Information Content per bp: | 1.778 |
| Number of Target Sequences with motif | 104.0 |
| Percentage of Target Sequences with motif | 3.16% |
| Number of Background Sequences with motif | 88.4 |
| Percentage of Background Sequences with motif | 0.21% |
| Average Position of motif in Targets | 108.7 +/- 25.7bp |
| Average Position of motif in Background | 106.0 +/- 68.1bp |
| Strand Bias (log2 ratio + to - strand density) | 0.7 |
| Multiplicity (# of sites on avg that occur together) | 1.01 |
| Motif File: | file (matrix) reverse opposite |

### Similar de novo motifs found

|  |  |  |  |  |  |  |  |
| --- | --- | --- | --- | --- | --- | --- | --- |
| Rank | Match Score | Redundant Motif | P-value | log P-value | % of Targets | % of Background | Motif file |
| 1 | 0.980 | G T C A A T G C G T C A G A T C C G T A T G A C A G C T A G C T A G C T G A C T T G C A G C T A C G T A | 1e-60 | -139.483311 | 2.95% | 0.20% | motif file (matrix) |
| 2 | 0.918 | A G T C G T C A G T A C G C T A G T A C G A C T G A C T A G C T A G C T G T C A G T C A T C G A | 1e-58 | -133.853493 | 3.10% | 0.26% | motif file (matrix) |
| 3 | 0.919 | G A T C C T G A A G T C C G T A A G T C C G A T G A C T G A C T C G A T C G T A C G T A T C G A G T A C G T A C | 1e-52 | -121.529844 | 2.70% | 0.21% | motif file (matrix) |
| 4 | 0.775 | T G C A T G C A T G C A A T C G A C G T C T A G C A G T A C T G A C G T C T G A C T A G | 1e-46 | -107.868098 | 2.67% | 0.26% | motif file (matrix) |
| 5 | 0.856 | A T G C T C G A G T A C A G C T G A C T A C G T G A T C C G T A C G T A G T C A A T G C | 1e-43 | -100.277072 | 2.85% | 0.35% | motif file (matrix) |
| 6 | 0.787 | C G T A C G T A A C T G A G C T C T A G A C G T A C T G A C G T | 1e-26 | -60.147621 | 4.89% | 1.75% | motif file (matrix) |
